# The economic impact of organic production in Brazil: A study based on municipal production hotspots

**DOI:** 10.1371/journal.pone.0264095

**Published:** 2022-03-09

**Authors:** Camila de Moura Vogt, Douglas Alcantara Alencar, Adelar Fochezatto

**Affiliations:** 1 Departamento de Economia–Instituto de Ciências Aplicadas–ICSA, Universidade Federal do Pará–UFPA, Belem, Brazil; 2 Departamento Programa de Pós-Graduação em Economia–PPGE, Escola de Negócios PUCRS, Pontifícia Universidade Católica do Rio Grande do Sul–PUCRS, Porto Alegre, Brazil; Szechenyi Istvan University: Szechenyi Istvan Egyetem, HUNGARY

## Abstract

The agroecological products market has increased substantially worldwide in recent decades. As a traditional agricultural country, Brazil has followed this trend and has increased the production of certified organic products in recent years. In addition, the country is one of the largest consumer markets in Latin America. This study aims to measure the effects of organic production on the economic development of municipalities through spatial analysis and econometric methodologies. Thus, it estimates the impact of organic production hotspots on the 2017 gross domestic product of Brazilian municipalities and the agriculture gross value added. The results indicate that the organic hotspots had a positive effect on both variables. Therefore, the results corroborate incentives for organic production as an alternative for the sustainable development of the agricultural sector.

## Introduction

Organic agriculture has increased its economic importance in the global agribusiness sector. In 2017, 69.8 million hectares of production were registered globally, which generated 97 billion U.S. dollars [1]. This represents an increase of 102% in the total cultivated area compared to 2008 and an increase of 93% in the total value traded. The number of producers also increased during this period, from 1.4 million producers in 2008 to 2.9 million in 2017.

In Brazil, the increase in the number of organic producer certifications and optimistic consumption forecasts reflect the growing importance of this segment for the country’s agribusiness sector [2]. Today, the country is the largest market for organic produce in Latin America and the Caribbean, according to 2017 projections [1].

However, although studies on the benefits of organic practices in relation to ecological issues are known, Brazilian organic production still needs to be evaluated regarding its effects on the economy and local development [3–5]. Furthermore, there is the recurrent question of the socio-economic advantages of investing in organic agriculture to the detriment of conventional production practices [6]. Thus, this study aims to assess whether the municipalities that excel in organic production have a higher economic effect on income growth than municipalities that have not followed the same path. Thus, the estimation of hotspots is used as an indication of specialization in organic production. Furthermore, it is understood that the reasons for locating these production sites in regions of greater concentration are linked to different characteristics, such as the better offer of specialized labor and technological know-how structures, in addition to logistical facilities [7].

Using data from the 2006 Agricultural Census and the 2017 Agricultural Census, released by the Brazilian Institute of Geography and Statistics (IBGE) [8–11], we estimate the economic effects on a municipality of having organic production hotspots. The propensity score model was used to assess the impact of organic production on the agriculture gross value added (GVA) and gross domestic product (GDP).Additionally, the effects are estimated for municipalities that did not previously have but developed organic agriculture hotspots between the last two censuses of 2006 and 2017.

### Organic production in Brazil

In Brazil, organic agriculture is characterized by production using specific techniques, the optimization of resources, and a respect for and the maintenance of cultural integrity of rural communities (Law n° 10.831). According to the 2017 Agricultural Census [11], Brazil has five million producers who dedicate their properties to conventional agriculture and 68,700 to organic production. Among the organic producers, 39,600 are devoted to crop production, 18,200 to animal production, and 10,800 to crop and animal production. Since 2006, however, the number of organic producers has not grown. The results of the 2006 Agricultural Census show that there were 90,400 organic producers in Brazil, 1.7% of the total.

Regarding their distribution in the states, most producers in 2006 [8] were located in Bahia, Minas Gerais, Rio Grande do Sul, and Paraná. In 2017 (IBGE, 2017), more than 50% of the producers were from Minas Gerais, Pernambuco, Paraná, São Paulo, and Rio Grande do Sul. The state of Bahia had the most significant reduction, reporting 13,600 fewer producers. Although there has been a drop in the number of properties that claim to be organic producers, there has been an increase in the number of certifications. In addition, there was also an increase in the number of organic manufacturing proprietors, corroborating the development of the organic production chain.

The reason for the decrease in the number of properties declared in the census but an increase in the number of certified properties is still unclear. However, it may be associated with an increase in information about organic techniques and the demand for certification labels by the consumer market. This may have led farmers to be more careful when defining themselves as organic properties, resulting in the fall in self-declared organic producers. The consumption of organic products, as well as certified organic production, also shows an expansionary forecast. The growing demand is supported by a series of studies that prove the benefits of organic consumption compared to products from conventional production [12].

According to the Brazilian Council for Sustainable Organic Production–Organis, in 2017, 15% of the consumer market regularly purchased organic products in the capitals, and projections indicate growth for the sector in the coming years [2]. Thus, studies that evaluate the economic effects of organic consumption have also been expanded, as well as the dissemination of the environmental benefits and an improvement in product distribution networks.

A milestone for the trade development of certified organic products is the National Policy on Agroecology and Organic Production (Pnapo) [1]. One of the policies is the expansion of the participation of organic products in purchases made by the Companhia Nacional de Abastecimento (Conab) in the Food Acquisition Program (PAA).

Although the adoption of organic production certification has shown growth, it still presents essential barriers to entry [13]. It mainly impacts the adoption of organic agriculture by business or agroindustrial producers [6]. It is known that the adoption of organic agriculture by farms is difficult due to the initial loss of productivity with agroecological production. If producers have a strong interaction with the market, the conversion to crops outside the "Green Revolution" package does not deliver results related to initial productivity gains [14]. Thus, the conversion of traditional agriculture to organic agriculture on a large scale does not occur regularly.

In contrast, due to the lower need to meet market demand, family farming has a lower cost of conversion to organic production. Thus, it facilitates the adoption of agroecological practices by agricultural family farm properties. Also, it is possible to observe the family farm structure and production with associations and cooperative schemes [15, 16].

As family farming represents lower production gains than conventional farming, the impact of organic farming on the socio-economic development of Brazilian municipalities is usually considered irrelevant. However, it is known that studies evaluating the economic results of organic production show that the economic effects are positive in several countries. Organic farmers have access to a premium market, which, despite not indicating more significant gains than conventional activities, due to costs, suggests the market’s potential, given its tendency to expand [17, 18]. Results also show that clusters of organic production present economic results that are superior to those of conventional production for reducing poverty and improving income indicators [19]. Thus, there is a consensus about the potential of organic output for economic development [20, 21]

Finally, based on the methodologies and variables used in studies to assess socio-economic impacts, we will evaluate the adoption and expansion of organic production in Brazilian municipalities [19]. For this, the study will focus on variables that can be considered proxies for municipalities’ rural economic development: GDP and agriculture: GAV.

## Materials and methods

To measure the economic effects of organic production, the municipalities were divided into two groups: those that are hotspots of organic production, the treatment group, and those that are not, the control group.

Thus, to identify organic production hotspots, methodologies were chosen that take into account spatial dependence. When observations are not spatially independent, if there is a relationship between the behavior of a variable associated with the same behavior of a nearby location (neighbor), then there will be spatial dependence or autocorrelation.

The first work to address spatial association and to construct a dependency statistic was by Moran [22]. Subsequently, Anselin [23] proposed a set of methods that sought to overcome the difficulties encountered by the first studies of spatial association. The model, called Local Indicators of Spatial Association (LISA), presents two essential characteristics, described by Simões [24]. First, the indicator’s value allows the inference of the statistical significance of the pattern of spatial association in the specific location, and the sum of local indicators of spatial association of all categories is proportional.

The definition of the neighborhood to be considered, or matrix of weights, is another critical point of the analysis. Whether due to contiguity, travel time, or economic distance, it is essential and deserves attention in formatting the models. In other words, such models make it possible to verify whether the presence of a phenomenon in an area makes its existence more or less likely in neighboring regions.

According to Simões [24], if there is a change in probability due to spatial proximity, there is spatial autocorrelation. It can be larger or smaller than a random pattern. If greater that the standard, it would characterize the formation of a cluster or clusters, at the limit, would lead to regular alternation in which the presence of a phenomenon in a region decreases (or eliminates) the probability of this same phenomenon in a contiguous (neighboring) area.

Moran’s I tests the simple linear global association between *y*_*i*_ and the spatial lag operator *W*_*i*_ (Eq 1):

$$I=\frac{n\sum_{j=1}^{n}\sum_{j=1}^{n}w_{ij}(y_{i}-\bar{Y})(y_{j}-\bar{Y})}{\sum_{j=1}^{n}w_{ij}\sum_{i=1}^{n}{\left(y_{i}-\bar{Y}\right)}^{2}}$$ (1)

where *y*_*i*_ and *y*_*j*_ are the observed *y* values in the region. *W*_*i*_ represents the matrix of weights under the null hypothesis of spatial non-correlation; the expected value of the indicator is given by $E(I)=\frac{-1}{\left(n-1\right)}$. A positive (negative) and statistically significant value indicates the presence of positive (negative) spatial dependence.

The LISA indicator shows the local spatial dependence (identifies the regions where the variable in question is spatially correlated). The numerator of the Moran’s I equation is decomposed for each of the areas. It is defined by (Eq 2):

$$I=z_{i}\sum_{j=1}^{n}w_{ij}z_{j}$$ (2)

where *z*_*j*_ is equal to the variable in region *j* minus the mean and *z*_*i*_ is equal to the variable in region *i* minus the mean. Considering Moran’s I and LISA together, two applications can be highlighted: the Moran scatter plot (scatter diagram) and the LISA cluster map. The first allows the identification of outliers (with significant influence on Moran’s I), and the second allows the detection of regions where the correlation is strong (clusters). Furthermore, it is possible to find low values for global autocorrelation and high values for local autocorrelation (in some places that form the set).

Thus, municipalities with relevant products were evaluated in the national context. Spatial dependency classifications high–high, high–low, and low–high were considered hotspots.

As organic production may be associated with logistics, proximity to the consumer market, characteristics of production and producers, or some other specific incentive [13], we cannot say that there is randomness in the presence of organic properties. Thus, it was necessary to pair the municipalities of the two groups in terms of observable characteristics to make them as similar as possible. In other words, to be observed as a valid control group, it is necessary to make a pairing such that the hypothesis of conditional independence is respected. The hypothesis supports that given a set of characteristics *X*, the attribution of treatment is unconditional to the potential outcome. Thus, once we control for *X*, treatment allocation to municipalities with a group of similar characteristics can be considered random [25].

It was possible to observe municipalities with similar characteristics of both the treatment and control groups during the pairing. One is thus counterfactual, since it is impossible to follow the same city in two different situations. In addition, the validity of the stable unit treatment value assumption (SUTVA) hypothesis is required, so the treatment received by a municipality does not affect municipalities with below-average production (untreated).

The presence of spatial dependence makes the ordinary least squares (OLS) estimation inappropriate, because the estimates will be biased, inconsistent, or inefficient. Therefore, spatial association models like LISA allow us to visualize patterns and describe regions and potential spillovers between areas through specific estimates for this situation.

The methodology used to perform the monitoring is propensity score matching (PSM), that is, a conditional probability *p*(*x*) of a municipality to be treated given a set of observable characteristics *x*.

p(x)=P(T=1|x) (3)

where *p*(*x*) is the propensity score, or the treatment probability *T* = 1 [26]. The set of variables *x* should not have been affected by the treatment, and therefore, ideally, pre-treatment characteristics should be used.

Thus, it is possible to evaluate the municipalities from the same initial characteristics. On the probability estimates *p*(*x*), the *probit* binary model is used (Eq 4).

$$p_{i}=P(Y=1|X=x_{i})=\phi (\beta_{0}+\beta x_{i})$$ (4)

where, *Y*_i_ assumes a binary value considering the presence or not of treatment, and *x*_*i*_ represents observable characteristics that will affect *p*_*i*_. That is, the probability considering *Y* = 1.

The literature documents some techniques for performing propensity score matching. These techniques aim to minimize selection bias and avoid inappropriate matches. Briefly, Cameron and Trivedi [27], together with Caliendo and Kopeinig [28], described four frequently used techniques. The first is nearest-neighbor (NN), which deals with choosing an individual from the comparison group as a corresponding partner for a treated individual that is closest in terms of propensity score. This choice can be made "with replacement" and "without replacement." Through this technique, the nearest non-participant *j* is selected for each municipality participating *i*. The choice with replacement reduces the number of distinct non-participants used to build the counterfactual result and, thus, increases the estimator’s variance.

Another technique is Caliper and Radius Matching (CRM), which is an extension of NN. All participating *i* and non-participating municipalities *j* with an estimated propensity score that fits the caliper are chosen. Stratification and Interval Matching (SIM) can also be used, which proposes to divide the propensity score into a set of intervals (strata) and calculate the impact on each interval, taking the average difference of the results between treatments and observations of control. Finally, there is Kernel and Local Linear Matching, where all treatment units are matched with a weighted average of all control units, according to the weights (inversely proportional) concerning the distance between the propensity score values of the treated and untreated groups.

Thus, it is possible to estimate the mean treatment effect (ATE) after pairing according to the selected technique. It is also possible to use weighted statistics, given by the inverse of the probability estimated by the propensity score if the municipality belongs to the treatment group and by the complement of this probability if the city belongs to the control group.

Beyond the ATE analysis, the difference-in-differences methodology can increase the robustness of the results. For analysis, the data set is verified before and after treatment, both for the treatment and control groups. The method compares the results of the municipalities after the treatment with their previous results. It reduces the outcome of the difference before and after those not treated, as shown in Table 1 below:

**Table 1:** Difference-in-differences.

	Census 2006	Census 2017	Differences
**Control**	↓% organic: A	↓% organic: C	C-A
**Treatment**	↓% organic: B	↑% organic: D	D-B
**Difference**	B-A	C-D	(D-B)-(C-A)

Source: Authors.

The method was used by Heckman *et al*. [29]; it identifies in a non-parametric way the estimator of a given program’s impact on those who participated (ATT). For example, the difference-in-differences model will consider the following equation (Eq 5):

$$Y_{it}=\alpha +\gamma_{1}{trat}_{it}+\gamma_{2}{censo}_{it}+\gamma_{3}{DD}_{it}+u_{it}$$ (5)

The *trat*_*it*_ is the treatment, *censo*_*it*_ is the dummy that identifies the Agricultural Census period (2006 or 2017), and finally *DD*_*it*_ that indicates the interaction *trat*_it_*censo_*it*_. The variable *u*_*it*_ indicates the error term. Regarding the estimators, *α* is a constant that shows the average estimate effect on the control group in the first period and *γ*_1_+*α* shows the average effect on the control group in the next period, *γ*_2_ is the difference between the control group and the treated group, and finally, the estimated *γ*_3_ shows the difference-in-differences effect, or the treatment effect, considering the difference between the two groups in both periods.

Ordinary least squares (OLS) (pooling) and, for more robust results, also fixed-effects estimates are considered for the results. The fixed-effects estimation of the first differences allows the observed variables to be arbitrarily correlated with the effects of the model’s unobserved variables [26].

The propensity score method is also associated with difference-in-differences in both OLS and fixed-effects estimates. Therefore, it is the most robust and reliable method to verify the impact of treatment on the population analyzed. In addition, it allows the weighting of the variables to avoid bias due to specification errors potentially present in a conventional fixed-effects regression. The procedure, called the Doubly Robust Estimator [30], consists of estimating a regression whose variables are weighted by the probabilities of receiving the treatment, previously calculated via the propensity score.

According to Imbens and Wooldridge [31], using the estimated probabilities to weight the variables in a fixed-effects estimation improves the robustness of the results, allowing for the elimination of biases of the omitted variable.

The municipal GDP and agriculture GVA were used for the evaluation of economic results. For the propensity score matching, the variables related to income, type, and the average size of properties and location characteristics were used (Table 2).

**Table 2:** Variables selected to assess the economic effect of organic production.

Variable	Descrition	Effect
**Organic Hotspot**	Treatment variable for organic production (treated, or production hotspot = 1, untreated = 0). Agricultural Census 2006 and 2017.	The municipalities were selected according to the spatial dependence of organic production, number of farms that indicated hotspots: high–high, high–low, and low–high.
**Average Farm Income 2006**	Average income received per establishment. 2006 Agricultural Census	Income characteristics of municipalities before 2016. It is expected that there will be a positive relationship between income and welfare characteristics such as education and self-financing and the organic production initiative [13].
**Average Rural Area 2006**	Average rural area per establishment. 2006 Agricultural Census	Land ownership characteristics of municipalities before the year 2016. The size of properties is expected to affect the adoption of agricultural practices [18].
**Family Farm Percentage 2006**	The proportion of family farms in the municipality. 2006 Agricultural Census	
**Gross Domestic Product (GDP) and Agriculture Gross Value Added (GVA)**	Municipal accounting IBGE 2006 and 2016	Organic agriculture is expected to have positive economic results for the variables [19].

Source: Authors.

Therefore, by considering studies evaluating the effects of organic agriculture on economic development, the results for the Brazilian market are estimated.

## Results and discussion

There were 5,570 Brazilian municipalities with an annual average GDP of R$ 1,181,888,000 in 2017. The agriculture GVA was R$ 54,373,000. These municipalities had an average of approximately 16 organic properties in 2006 and 12 in 2017. However, the number of non-organic properties was considerably higher, from around 913 to 899, respectively, in the two years.

**Fig 1:**
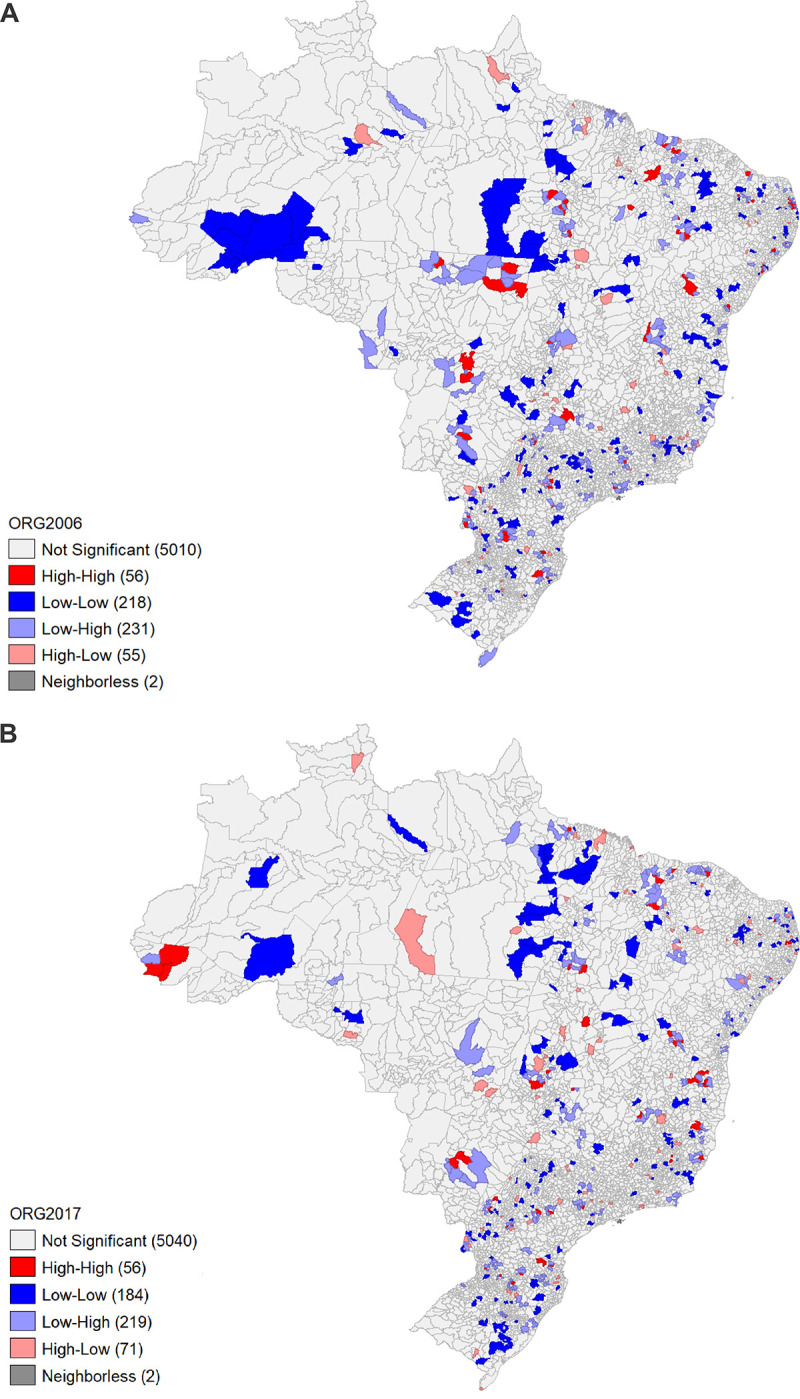
Map showing the LISA indicator of spatial dependence for Brazil in 2017 (map above) and 2006 (map below). Source: Calculated by the authors. Software GEODA.

Fig 1(A) shows the LISA indicators for Brazilian municipalities in 2017 (upper map) and 2006 (lower map). In 2017, 56 municipalities had a high–high dependency ratio as a function of the number of establishments with organic production, 219 low–high, 71 high–low, and 184 low–low, and 5,040 municipalities did not present a significant relationship. Thus, 346 municipalities were considered organic production hotspots. In 2006 (B), 56 municipalities had a high–high dependency ratio as a function of the number of establishments with organic production, 231 low–high, 55 high–low, and 218 low–low, and 5,010 municipalities did not present a significant relationship. Thus, 342 municipalities were considered organic production hotspots.

The *probit model* results showed a negative and significant relationship with Average Farm Income in 2006 (Table 3). The average area and the percentage of family farms were not significant. However, it was decided to leave them in the statistics for pairing [32]. Thus, the existence or not of organic production hotspots is influenced by factors related to Brazilian municipalities’ economic, land tenure, and location characteristics. The characteristics previously mentioned are correlated with the organic producer’s profile [13].

**Table 3:** *Probit* results: Municipalities with organic production hotspots.

Variable	Coefficient
**Intercept**	−2.549*** (0.379)
**Average Farm Income 2006**	−0.000001* *(0*.*000)*
**Average Rural Area 2006**	−0.0001 *(0*.*0004)*
**Family Farm Percentage 2006**	−0.0766 *(0*.*440)*
**Number of obs**	5,547
**Pseudo R2**	0.0618

Source: Calculated by the authors. Signif. codes: 0 ‘***’ 0.001 ‘**’ 0.01 ‘*’ 0.05 ‘.’ 0.1 ‘ ’ 1.Standard error in parentheses. Software R Studio.

The results reinforce the need for pairing to estimate the economic effects of organic production. Therefore, considering that organic production is not a random event, a simple comparison between the municipalities with the largest producers and those without would present biased results.

Through a score ranging from 0 to 1, the propensity score represents the probability of each municipality receiving treatment. The variables of the comparison groups paired here did not necessarily have overlapping values; therefore, the RStudio software caliper optimization filter was used to perform the pairing, with a margin of 0.02 in the differences between the common support data. According to Austin [33], this is an adequate overlap between the propensity scores of the treatment and those of the control group.

Table 4 shows the average propensity score estimates between the control and treatment group municipalities for the unpaired and paired samples. The means presented show a standard mean difference of less than 1% in the propensity scores after matching, which indicates a considerable improvement in the possibility of comparison to verify the causality of the influence of organic hotspot production on the variables between the treated and non-treated municipalities.

**Table 4:** Matching results by propensity score (PSM).

Propensity score: unmatched sample control	Propensity score: unpaired sample treatment	Standard mean difference (%)	Propensity score: sample control with pairing	Propensity score: sample treatment with pairing	Standard mean difference (%)
0.0637	0.0625	0.2142	0.0637	0.0637	−0.0001

Source: Calculated by the authors. Software R Studio.

Taking as a sample a set of 694 municipalities (347 in each group), from the total 5,547 (municipalities with missing data and those that did not exist in 2006 were not considered in the sample), paired by Average Farm Income 2006, Average Rural Area 2006, and Family Farm Percentage 2006, Fig 2 presents the graphic distribution of propensity scores between paired and unpaired municipalities for the control and treatment samples.

**Fig 2:**
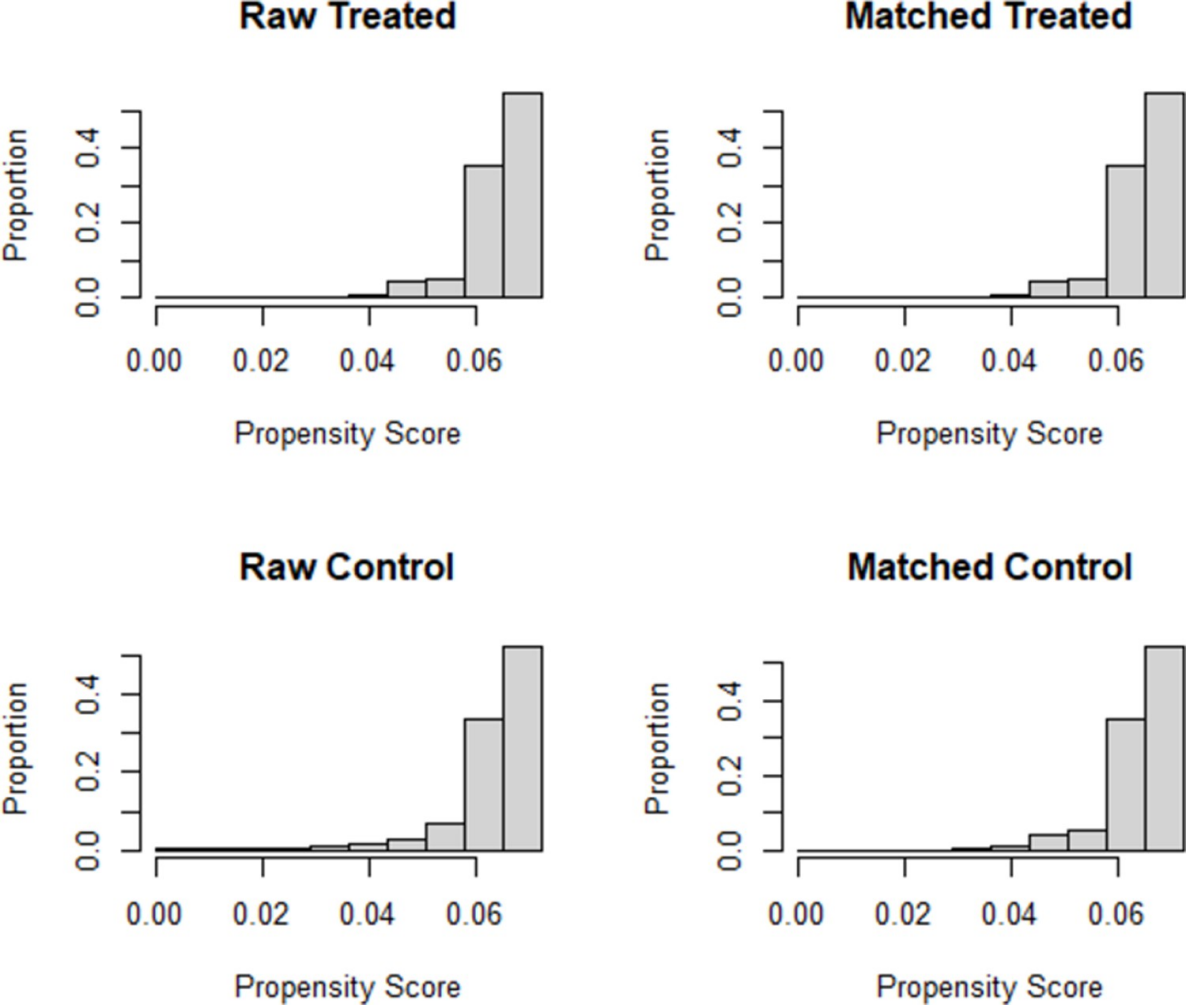
Graphs showing matching results by propensity score (PSM). Source: Calculated by the authors. Software R Studio.

The results relating to ATE show a positive difference in agriculture GVA and GDP after PSM. In Table 5, it is possible to verify that, according to the *t*-test, the values of GDP and agriculture GVA are not statistically significant before the pairing but are after. Thus, it is possible to affirm a positive difference between the treated municipalities and the control municipalities after the matching of R$657,818,000 for the GDP and R$13,046,000 for the agricultural GVA. However, the average agriculture GVA in 2017 behaves differently. Before pairing, the difference in means shows that municipalities in the control group have a value of R$ 25,000 more than the municipalities in the treatment group. However, after pairing, this difference is not significant.

**Table 5:** *t statistic* for paired and unpaired economic variables for the control and treatment groups.

Variable	Average Effect Unpaired	Valor-p	Average Effect Paired	Valor-p
GDP 2017 (R$ 1,000)	189,107.00	0.5569	657,818.00	0.04111*
Agriculture Gross Value Added 2017 (R$ 1,000)	2,709.80	0.5508	13,046.65	0.0185*
Average Agriculture Gross Value Added 2017 (R$ 1,000)	−24.47	0.0017**	2.3	0.01855

Source: Calculated by the authors. Signif. codes:0 ‘***’ 0.001 ‘**’ 0.01 ‘*’ 0.05 ‘.’ 0.1 ‘ ’ 1. Standard error in parentheses. Software R Studio.

In summary, ATE estimates show that the economic effect of organic production on municipalities is positive. In other words, organic production contributes to the expansion of the product and has a significant economic impact compared to the same municipalities with less production. The positive effect was already estimated for other countries such as the United States, the United Kingdom, and India [17–19]. Therefore, it can be concluded that encouraging organic production in the municipalities is a way to increase economic results.

The difference-in-differences for GDP and agriculture GVA show that, for the production variables, there is no significant difference between 2017 and 2006 for the organic production hotspots (Tables 6 and 7). The only significant effect is when considering the growth of hotspot municipalities over time: R$ 742,622,000 for GDP and R$ 35,481,000 for agricultural GVA. Furthermore, estimates are made using the OLS pooling, fixed-effects, and random-effects methodologies, of which, according to the Hausman test, the values for the fixed-effects estimates would be more efficient.

**Table 6:** Difference-in-differences GDP results: *Pooling, fixed-effects, and random-effects*.

	OLS (1)	FE (2)	RE (3)
**Treated**	−12,331.280	54,155.260	33,616.070
	(492,444.200)	(392,137.300)	(359,274.900)
**Time**	736,894.800***	742,622.300***	740,914.700***
	(173,072.800)	(99,654.230)	(98,898.640)
**DID**	201,437.800	108,967.900	136,545.200
	(694,549.300)	(529,998.400)	(492,338.200)
**Constant**	433,182.300***		430,345.700***
	(122,357.500)		(120,578.000)
**N observations**	11,144	11,144	11,144
**R^2^**	0.002	0.011	0.006
**F Statistic**	6.714***	21.223***	63.727***

Source: Calculated by the authors. Signif. codes:0 ‘***’ 0.001 ‘**’ 0.01 ‘*’ 0.05 ‘.’ 0.1 ‘ ’ 1. Standard error in parentheses. Software R Studio.

**Table 7:** Difference-in-differences agriculture GVA results: *Pooling, fixed-effects, and random-effects*.

	OLS (1)	FE (2)	RE (3)
**Treated**	1,321.975	2,453.394	1,841.164
	(3,845.165)	(4,034.983)	(3,429.995)
**Time**	35,389.220***	35,481.430***	35,432.810***
	(1,351.409)	(1,025.414)	(1,011.700)
**DID**	1,387.823	-101.522	683.878
	(5,423.267)	(5,453.535)	(4,747.295)
**Constant**	18,815.370***		18,783.320***
	(955.407)		(949.363)
**N observations**	11,144	11,144	11,144
**R^2^**	0.062	0.194	0.108
**F Statistic**	245.134***	448.060***	1,344.556***

Source: Calculated by the authors. Signif. codes:0 ‘***’ 0.001 ‘**’ 0.01 ‘*’ 0.05 ‘.’ 0.1 ‘ ’ 1. Standard error in parentheses. Software R Studio.

The average agriculture GVA, unlike the other variables, shows a significant result for the existence of organic production hotspots. As shown in Table 8, there is a significant positive effect over time for hotspots in municipalities, of R$ 68,000. However, there is a negative impact of R$ 25,000 when considering the effect of having an organic economy hotspot. In other words, having a production hotspot does not positively impact income growth when we consider the municipalities in the control group.

**Table 8:** Difference-in-differences average agriculture, GVA results: *Pooling, fixed-effects, and random-effects*.

	OLS (1)	FE (2)	RE (3)
**Treated**	−3.483	13.156	4.429
	(12.360)	(12.578)	(10.813)
**Time**	68.261***	68.820***	68.544***
	(4.344)	(3.197)	(3.157)
**DID**	−20.992	−30.120*	−25.601*
	(17.432)	(17.000)	(14.945)
**Constant**	43.123***		42.634***
	(3.071)		(3.049)
**N observations**	11,144	11,144	11,144
**R^2^**	0.023	0.082	0.043
**F Statistic**	85.765***	165.175***	496.453***

Source: Calculated by the authors. Signif. codes:0 ‘***’ 0.001 ‘**’ 0.01 ‘*’ 0.05 ‘.’ 0.1 ‘ ’ 1. Standard error in parentheses. Software R Studio.

Tables 9 and 10 show the estimates of difference-in-differences by aggregating the PSM statistic. The results confirm that there is no effect on the creation of hotspots between 2006 and 2017. However, there is still a positive effect on the income of municipalities with hotspots of R$ 709,240,000 for GDP and R$ 31,747,000 for agriculture GVA.

**Table 9:** Difference-in-differences with PSM weight GDP results: *Pooling, fixed-effects, and random-effects*.

	OLS (1)	FE (2)	RE (3)
**Treated**	5,890.781	18,615.600	15,023.720
	(493,680.500)	(393,073.300)	(360,264.500)
**Time**	706,943.000***	709,240.300***	708,554.800***
	(173,732.200)	(100,027.300)	(99,319.870)
**DID**	170,411.100	133,961.900	144,832.600
	(693,563.800)	(528,730.800)	(491,490.100)
**Constant**	414,924.800***		414,359.800***
	(122,788.400)		(120,954.300)
**N observations**	11,094	11,094	11,094
**R^2^**	0.002	0.011	0.006
**F Statistic**	6.115***	19.339***	58.009***

Source: Calculated by the authors. Signif. codes: 0 ‘***’ 0.001 ‘**’ 0.01 ‘*’ 0.05 ‘.’ 0.1 ‘ ’ 1. Standard error in parentheses. Software R Studio.

**Table 10:** Difference-in-differences with PSM weight agriculture GVA results: *Pooling, fixed-effects, and random-effects*.

	OLS (1)	FE (2)	RE (3)
**Treated**	903.061	821.380	895.038
	(3,279.950)	(3,430.327)	(2,922.510)
**Time**	31,664.080***	31,747.010***	31,703.310***
	(1,154.254)	(872.933)	(863.127)
**DID**	4,929.222	3,628.765	4,313.286
	(4,607.949)	(4,614.203)	(4,027.530)
**Constant**	17,115.580***		17,116.080***
	(815.791)		(809.793)
**N observations**	11,094	11,094	11,094
**R^2^**	0.069	0.195	0.108
**F Statistic**	274.110***	503.251***	1,504.816***

Source: Calculated by the authors. Signif. codes:0 ‘***’ 0.001 ‘**’ 0.01 ‘*’ 0.05 ‘.’ 0.1 ‘ ’ 1.Standard error in parentheses. Software R Studio.

For the average agriculture GVA, the results considering the weighting by PSM show that the effects for municipalities with hotspots are positive (Table 11). However, the effects between control and treatment groups over time are not significant.

**Table 11:** Difference-in-differences with PSM weight average agriculture GVA results: *Pooling, fixed-effects, and random-effects*.

	OLS (1)	FE (2)	RE (3)
**Treated**	−3.299	5.602	0.974
	(7.049)	(7.227)	(6.173)
**Time**	52.545***	52.846***	52.699***
	(2.480)	(1.839)	(1.802)
**DID**	−1.005	−5.989	−3.544
	(9.902)	(9.721)	(8.493)
**Constant**	31.677***		31.413***
	(1.753)		(1.747)
**N observations**	11,094	11,094	11,094
**R^2^**	0.041	0.093	0.049
**F Statistic**	159.373***	305.148***	929.569***

Source: Calculated by the authors. Signif. codes:0 ‘***’ 0.001 ‘**’ 0.01 ‘*’ 0.05 ‘.’ 0.1 ‘ ’ 1. Standard error in parentheses. Software R Studio.

In general, the results show that for the 2017 data, it is possible to verify a significant mean difference. Therefore, it indicates positive causality between the presence of hotspots and the economic production of the municipalities. However, when analyzing the trajectory over time between 2006 and 2017, it is not possible to assert causality between the hotspots and economic production in the municipalities. In other words, it can be said that Brazilian organic production hotspots can be considered as an option for economic development for the regions beyond the ecological benefits [34]. However, this effect may be recent, which justifies the results obtained.

## Conclusion

Participation in organic agriculture has increased; however, the economic effects of production are still unclear for socio-economic development in Brazil. Thus, this work has shown that municipalities that excelled in the cultivation of organic agriculture obtained positive economic results. Additionally, municipalities with higher organic production also had positive results. Therefore, the outcomes of this work can be used as a reference to justify more significant agricultural subsidies and incentives for organic production and show that the development of organic production hotspots has a positive impact on municipal revenue and, consequently, the socio-economic development of the region.

However, it is necessary to emphasize the limitations of this work, it is impossible to guarantee that localities can resell their products at different prices from conventional production. Additionally, It does not evaluate the effects of traditional farming with high added value. Hence, it cannot be concluded that organic production is superior to other non-organic production methods. To obtain results that guarantee the superiority of the economic benefits of organic agriculture over traditional agriculture, comparative studies should be done.

Finally, this work concludes that, despite the positive results, further investigation into the impacts of the production of organic products is still necessary. It would be interesting to use data from certified properties and the comparative evaluation of conventional and organic production hotspots for future studies.

## Supporting information

S1 Data Data base 01. Source: IBGE (2006, 2016 and 2017).
(XLSX)

S2 Data Data base 02. Source: IBGE (2006, 2016 and 2017).
(XLSX)

S3 Data Data base spatial estimates. Source: IBGE (2006, 2016 and 2017).
(ODS)

S1 File Weight matrix. (GAL)
